# Characteristics and Prognostic Markers of Aggressive Subtypes of Thyroid Cancer: A Retrospective Study

**DOI:** 10.1002/cnr2.70131

**Published:** 2025-03-14

**Authors:** Suhaib Radi, Mazin Al‐Maghrabi, Saleh Binmahfooz, Miguel Franco, Richard Payne, Michael Tamilia

**Affiliations:** ^1^ College of Medicine King Saud Bin Abdulaziz University for Health Sciences Jeddah Saudi Arabia; ^2^ King Abdullah International Medical Research Centre Jeddah Saudi Arabia; ^3^ Department of Internal Medicine, Division of Endocrinology Ministry of the National Guard‐Health Affairs Jeddah Saudi Arabia; ^4^ Division of Endocrinology and Metabolism Jewish General Hospital, McGill University Montreal Quebec Canada; ^5^ Faculty of Medicine King Abdulaziz University Jeddah Saudi Arabia; ^6^ Academic Unit of Medicine Autonomous University of Nayarit Tepic Nayarit Mexico; ^7^ Department of Otolaryngology Royal Victoria Hospital, McGill University Montreal Quebec Canada

**Keywords:** BRAF, Ki‐67, poorly differentiated thyroid cancer, thyroid cancer aggressive variant of papillary thyroid cancer

## Abstract

**Objective:**

The prevalence of thyroid cancer has increased significantly. Aggressive subtypes of papillary thyroid cancer (AG‐PTC) and high‐grade follicular cell‐derived malignancies (HGFM) are malignancies that lie between well‐differentiated and undifferentiated cancers, and their management needs to be clarified. The aim of our study is to describe the clinicopathological characteristics of AG‐PTC and HGFM and to assess their prognostic value.

**Methods:**

This was a retrospective chart review study at single center of patients with AG‐PTC or HGFM. HGFM comprised of patients with poorly differentiated thyroid cancer (PDTC) and differentiated high‐grade thyroid carcinoma. The clinical presentation, pathological characteristics, molecular markers, specific treatments, and clinical outcomes were compared between the groups.

**Results:**

Of the 3244 thyroid cancer charts reviewed, 136 met the criteria for AG‐PTC and HGFM. The mean age at diagnosis was 49 years, with a predominance of women. The median follow‐up duration was 3 years. The rate of persistent or recurrent disease was 40.3% in the AG‐PTC group and 29.3% in the HGFM group, 4.5% died in the AG‐PTC group, and 1.8% died in the HGFM group. The presence of vascular, lymphovascular invasion and extrathyroidal extension were associated with a higher incidence of persistent or recurrent disease (Hazard ratio: 2.5, 3.8, and 4.2, respectively; *p* < 0.05). When the Ki‐67 index was divided into five groups, the recurrence rate was higher in the ≥ 20% Ki‐67 group.

**Conclusions:**

Possible prognostic markers for predicting worse prognosis include vascular/lymphovascular invasion, extrathyroidal extension, and the proliferative index Ki‐67.

## 1 Introduction

Thyroid cancer is the most prevalent endocrine tumor, and its prevalence has increased significantly over the last 20 years owing primarily to the increased detection of thyroid microcarcinomas [1]. It is estimated that thyroid cancer constitutes around 2% of all malignancies worldwide [1]. Follicular cell‐derived thyroid cancer includes different histologic types: papillary thyroid cancer (PTC), which is the most common type; follicular thyroid cancer (FTC), poorly differentiated thyroid cancer (PDTC), and anaplastic thyroid cancer (ATC). PTC and FTC are usually well differentiated and have good prognoses. In contrast, PDTC and ATC are rarer, dedifferentiated, and have worse outcomes [2, 3].

The aggressive subtypes of PTC (AG‐PTC) and the high‐grade follicular cell‐derived malignancies (HGFM); including PDTC are malignancies that lie between well‐differentiated and undifferentiated anaplastic cancers. The features and management of well‐differentiated cancers have been established in the literature; however, AG‐PTC and HGFM need to be clarified further because they have characteristics different from those of their more benign counterparts [4]. The prevalence of AG‐PTC and PDTC is relatively low; however, the incidence of persistent or recurrent disease at 1 year after primary therapy is high compared with that of the more well‐differentiated tumors [5]. For example, the locoregional and distant control for differentiated thyroid cancers is more than 90% compared to around 60%–80% in poorly differentiated thyroid cancer [5]. Furthermore, AG‐PTC subtypes possess distinct features that distinguish it from well‐differentiated PTC with a higher rate of distant metastasis (7%–11% vs. 4%) and lower avidity to radioactive iodine (RAI), as up to 20% of tall‐cell variants are refractory to RAI [6]. The 5‐year survival rate for thyroid cancer varies according to histotype; ranging from 63.6% for PDTC up to 91.1% for PTC [7]. Although PTC has a good prognosis; there are aggressive subtypes with clinicopathological and genomic backgrounds different from those of the classical PTC with worse prognosis [8]. The 5‐year survival in some of the AG‐PTC can be as low as 65% compared to > 90% in PTC [6, 8].

The development of molecular techniques has led us to identify clinically significant biomarkers useful in the diagnosis and prognosis of PTC [9]. Knowing the features and markers that predict a worse prognosis can help treat and monitor patients after primary therapy. The *BRAF*‐encoded BRAF protein sends signals to direct cell proliferation [10]. BRAF mutations are associated with more invasive disease and a higher risk of recurrence in patients with thyroid cancer compared to those without BRAF mutations [11]. However, whether BRAF mutations are associated with HGFM remains unclear [12].

The Ki‐67 index is a widely used biomarker for assessing the cell proliferation rate of certain cancers, such as breast cancer and neuroendocrine neoplasia [13, 14]. Ki‐67 expression and its role in thyroid cancer have been debated; however, it has potential implications for differentiating between benign and malignant tumors [15]. Ito et al. found that the proliferative index Ki‐67 is an independent prognostic factor for disease‐free survival in patients with PTC [16]. Furthermore, a high Ki‐67 index in patients with PTC is associated with higher recurrence rates and poor survival [16, 17].

In the present study, we aimed to analyze and characterize the clinical, pathological, and genotypic aspects of AG‐PTC and HGFM and their prognostic significance in this patient cohort. We primarily aimed to determine the association of the Ki‐67 index and *BRAF* mutations with recurrent or persistent thyroid cancer. This may contribute to directing surveillance and individualizing the therapeutic strategies in such patients.

## 2 Materials and Methods

### 2.1 Study Design, Setting, and Population

After obtaining ethics approval from the Research Ethics Board (REB), project number 2020‐1902, this retrospective chart review of adult patients of both sexes, aged ≥ 18 years, with thyroid cancer over the last 10 years, between 2010 and 2019 at the Jewish General Hospital in Montreal, Canada, affiliated with McGill University, was conducted. A total of 3324 medical records were reviewed, and patients with AG‐PTC or PDTC were included. Patients with other types of thyroid cancer were excluded, leaving 136 patients who met the diagnostic criteria. Of the total cohort, 124 patients were followed up for ≥ 1 year.

### 2.2 Definitions of AG‐PTC and PDTC and Other Variables

According to the World Health Organization (WHO) criteria, AG‐PTC is defined as the presence of one of the following histologic patterns: tall cell, clear cell, diffuse sclerosing, columnar cell, or hobnail [18]. The presence of one or more of these patterns at ≥ 30% is considered a subtype; otherwise, it would be called “features.” The recently released 2022 WHO report distinguishes the two types of high‐grade follicular cell‐derived malignancies (HGFM): PDTC and differentiated high‐grade thyroid carcinoma (DHGTC) [18]. PDTC was defined using the Turin criteria as the presence of a solid/trabecular pattern, an insular growth pattern, and one of three high‐grade features—mitosis (> 3/10 high‐power field), necrosis, or convoluted nuclei. DHGTC was defined based on high mitotic activity and tumor necrosis, regardless of histological differentiation. Both types were grouped together in the present study under HGFM, considering similar biologic behaviors [18]. Regarding staging, we used TNM classification from the American Joint Academy on Cancer (AJCC) 7th (for patients diagnosed before 2016) and 8th editions (for patients diagnosed from 2016 onwards).

### 2.3 Outcomes

Response to therapy was defined based on the American Thyroid Association criteria. Excellent response was defined as absence of clinical, biochemical, and radiological disease [19]. Persistent disease was defined as an incomplete response, either structural (persistent loco‐regional or distant metastasis) or biochemical (abnormal thyroglobulin or rising thyroglobulin antibodies in the absence of localizable disease) [19]. Initial response to therapy was assessed at 12 months after RAI (or after surgery in those who did not receive RAI). Recurrence was defined as evidence of disease confirmed using either structural or functional imaging or biopsy after 6 months of being disease‐free. Disease‐free survival was defined as the duration during which a patient remains free of any evidence of disease, either biochemically or radiologically, after completing the treatment, indicating the absence of disease recurrence or progression during that period. Ki‐67 index and BRAF mutations were included in only some of the medical records reviewed because they were routinely analyzed more frequently in all aggressive types recently and much less frequently in older specimens. Ki67 labeling index was defined as the percentage of immunopositive tumor cells on nuclear staining in hotspot areas of 500–2000 tumor cells.

### 2.4 Statistical Analysis

The characteristics of patients and tumors were compared between those with and without evidence of persistent disease, recurrence, or death using the Pearson *x* ^2^ test. Univariate and multivariate analyses were performed using the log‐rank test and the Cox proportional hazards method, respectively. Statistical significance was set at *p* < 0.05. Statistical analysis was performed using SPSS version 26.0 (IBM).

## 3 Results

In total, out of the 3324 patients screened, 136 patients were included in this study: 74 in the AG‐PTC group and 62 in the HGFM group. This constitutes a prevalence of AG‐PTC of 2.2% and HGFM of 1.9%. Furthermore, most patients were females in the AG‐PTC (70.30% [*n* = 52/74]) and HGFM (59.7% [*n* = 37/62]) groups. The mean age was 44 (+/− 18.7) and 54 (+/− 14.5) years in the AG‐PTC and HGFM groups, respectively (*p* = 0.0005; Table 1). Mean follow‐up was 4.52 years in the AG‐PTC group and 4.89 years in the HGFM group.

**TABLE 1:** Baseline characteristics.

	AG‐PTC (*n* = 74)	HGFM (*n* = 62)	*p*
Mean (+/− SD) age in years *	44 +/− 18.7	54 +/− 14.5	0.0005
Female	70.3%	59.7%	0.03
Mean (+/− SD) f/u in years *	4.52 +/− 5.04	4.89 +/− 5.12	0.07
Hobnail features	12	5	NA
Diffuse sclerosing features	26	1	
Columnar features	3	3	
Tall cell features	41	4	
Clear cell features	2	1	
Insular features	0	7	
Mitosis	0	5	
Necrosis	0	10	
Solid features	0	38	
Trabecular features	0	27	
Convoluted nuclei features	0	1	
> = 30% involvement	44.6%	62.9%	0.06
Mean (+/− SD) tumor size in cm *	2.71 +/− 1.6	3.58 +/− 2.25	0.0133
T3/T4 stage	54.8%	37.1%	0.03
Extrathyroidal extension	51.4%	21%	0.0039
Vascular invasion	23%	21%	0.591
Lymphovascular invasion	71.6%	40.3%	0.000087
Positive margins	40.5%	29%	0.514
pN1 stage	75.3%	29%	< 0.005
pM1 stage	8.3%	12.9%	0.231
Biochemical response Complete Partial Indeterminate	Out of 65 49.2% 32.3% 18.5%	Out of 54 72% 22.2% 5.6%	0.022
Structural response Complete Partial Indeterminate	Out of 66 60.6% 19.7% 19.7%	Out of 53 66% 17% 17%	0.83
Mean (+/− SD) Ki67 *	5.42 +/− 4.06	12.23 +/− 12.03	0.0033
Ki67 groups < 10 > = 10	Out of 24 75% 25%	Out of 35 45.7% 54.3%	0.024
BRAF positive	84.6% (out of 26)	16.7% (out of 24)	< 0.005

Abbreviations: AG‐PTC: aggressive‐papillary thyroid cancer; HGFM: high‐grade follicular cell‐derived malignancies; pM1: distant metastasis; pN1: lymph node metastasis; SD: standard deviation.

* Statistical method used was Mann–Whitney U test.

The most prominent subtype in the AG‐PTC group was tall‐cell (55.4%, *n* = 41/74), followed by diffuse sclerosing (35.1%, *n* = 26/74) and hobnail (16.2%, *n* = 12/74). The most common growth pattern in the HGFM group was solid (61.3%, *n* = 38/62), followed by trabecular (43.6%, *n* = 27/62). The mean tumor size was 2.71 cm (+/− 1.60) and 3.58 cm (+/− 2.25) in the AG‐PTC and HGFM groups, respectively (*p* = 0.0133). Furthermore, more patients had advanced disease (stages T3 and T4) in the AG‐PTC group than in the HGFM group (54.8% vs. 37.1%, *p* = 0.03). Extrathyroidal extension occurred in more patients in the AG‐PTC group than in the HGFM group (51.4% vs. 21%, *p* = 0.0039). Similarly, more patients had lymphovascular invasion (71.6%) in the AG‐PTC group than in the HGFM group (40.3%) (*p* < 0.005). The rest of tumor pathological characteristics are presented in Table 1.

Six patients had metastasis on presentation in the AG‐PTC group, whereas eight had metastasis in the HGFM group. Of the eight patients with pM1 on presentation (four biopsy‐proven, three to the lungs, and five to the bones) in the HGFM group, five were RAI‐avid, two were flurodeoxyglucose (FDG)‐avid, and one didn't show uptake to RAI or FDG. Of the six patients with pM1 on presentation (four biopsy‐proven, three to the lungs, and three to the bones and lungs) in the AG‐PTC group, five were RAI‐avid and one was FDG‐avid.

Furthermore, 89.2% of patients received RAI treatment in the AG‐PTC group, whereas 74.19% received it in the HGFM group. Two patients underwent radiotherapy in the AG‐PTC group, whereas one underwent radiotherapy in the HGFM group. Two of the three patients with FDG‐avid disease received RAI treatment. Central neck dissection was performed in 61.81% and 68% of patients in the AG‐PTC and HGFM groups, respectively. Furthermore, 32.43% and 11.29% of patients underwent central and lateral neck dissections in the AG‐PTC and HGFM groups, respectively. Lobectomy only was performed in 4% and 16.13% of patients in the AG‐PTC and HGFM groups, respectively. The reason for performing lobectomy in most cases was the aggressive nature of the disease, which made total thyroidectomy unfeasible.

Notably, more patients achieved complete biochemical response in the HGFM group than in the AG‐PTC group (72% [*n* = 39/54] vs. 49.2% [*n* = 32/65]), *p* = 0.022. The complete structural responses of both groups were similar (AG‐PTC: 60.6% [*n* = 40/66], HGFM: 66% [*n* = 35/53]).

In the AG‐PTC group, 19.4% of patients had persistent disease, whereas 29.9% had recurrent disease. In the HGFM group, 15.5% of patients had persistent disease, whereas 22.4% had recurrent disease. The combined persistent or recurrent disease rate was numerically higher in the AG‐PTC group than in the HGFM group (40.3% vs. 29.3%, *p* = 0.137).

Of 13 patients with recurrent HGFM, 11 had locoregional disease. In contrast, all 20 patients with recurrent AG‐PTC had locoregional disease (*p* = 0.148). Eight of the 13 patients with recurrent HGFM had distant metastasis, whereas seven of the 20 patients with recurrent AG‐PTC had distant metastasis (*p* = 0.128). Four patients died during the follow‐up period: three in the AG‐PTC group and one in the HGFM group (Table 2).

**TABLE 2:** Prognosis of AG‐PTC and HGFM according to recurrence, persistence, and mortality rates.

Outcomes	AG‐PTC (*n* = 74)	HGFM (*n* = 62)	*p*
Persistent disease	19.4% (out of 67)	15.5% (out of 58)	0.371
Recurrent disease	29.9% (out of 67)	22.4% (out of 58)	0.231
Persistent/recurrent disease	40.3% (out of 67)	29.3% (out of 58)	0.137
Mean (+/− SD) time till recurrence in years *	2.71 +/− 3.05	4.23 +/− 3.81	0.2395
Mean (+/− SD) number of recurrences *	1.6 +/− 0.75	1.62 +/− 0.96	0.9615
Death	4.5% (3 out of 67)	1.8% (1 out of 56)	0.38
Mean (+/− SD) duration till death (in years)	4 +/− 3	2	

Abbreviations: AG‐PTC: aggressive‐papillary thyroid cancer; HGFM: high‐grade follicular cell‐derived malignancies; SD: standard deviation.

* Statistical method used was Mann–Whitney U test.

Since our study was retrospective, Ki67 and BRAF testing was not available for all patients and was mostly present for those diagnosed in the last 3 years of assessment. Out of total cohort, Ki67 testing was performed on 59 subjects and BRAF testing was available for 50 patients. Although there was a numerical increase in the rate of persistent/recurrent disease among BRAF‐mutated patients, it did not reach statistical significance. Also, there was no difference between high (≥ 10%) and low (< 10%) Ki‐67 groups. Similarly, no statistically significant difference was observed between patients with ≥ 30% involvement of their aggressive or poorly differentiated features and those with less involvement. When the Ki‐67 index was divided into five groups, the recurrence rate was higher in the ≥ 20% Ki‐67 group (five patients vs. 1, *p*‐value of 0.017).

**FIGURE 1:**
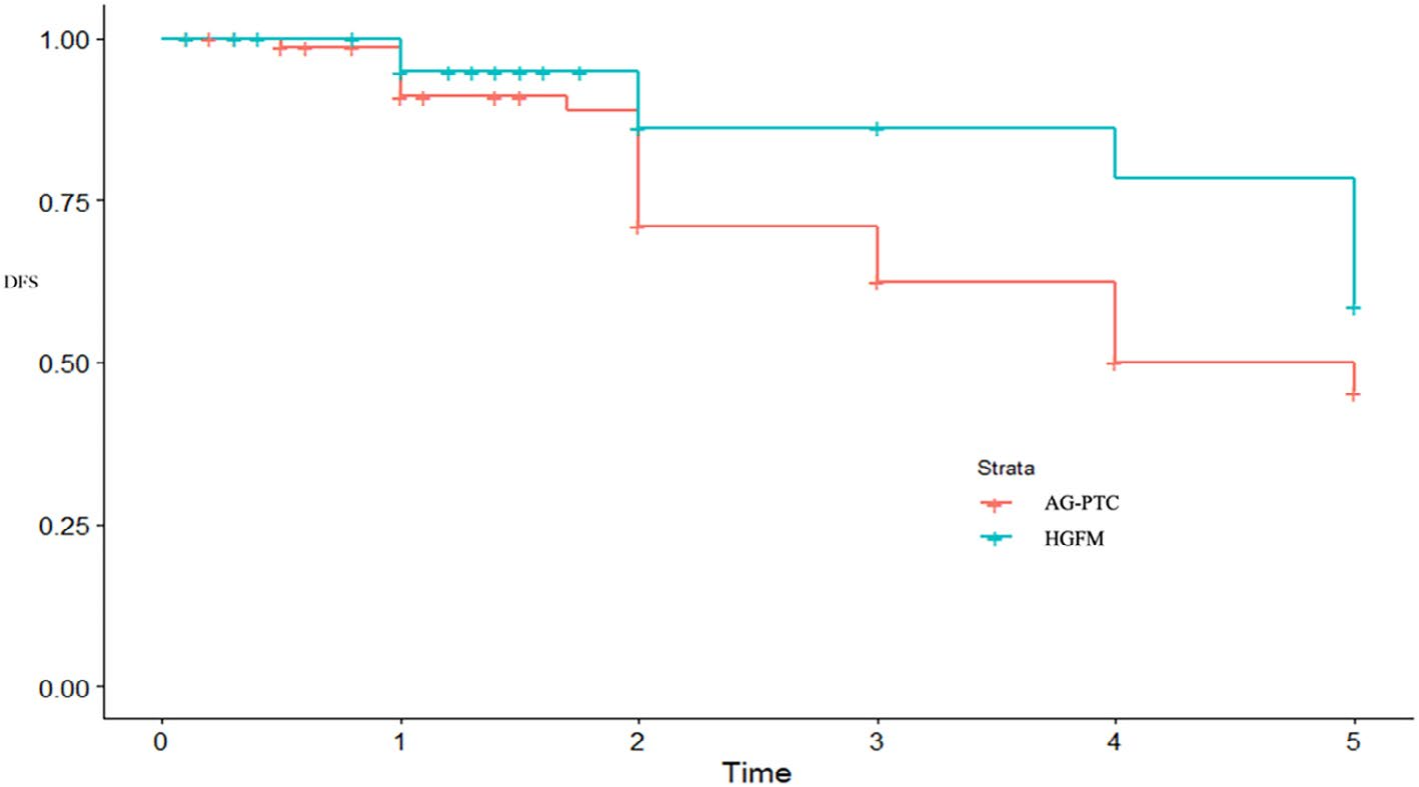
Kaplan–Meier curve for disease‐free survival in the AG‐PTC and HGFM groups. Disease‐free survival was defined as absence of any evidence of disease. So both persistent disease and recurrent disease were considered as events. AG‐PTC: aggressive‐papillary thyroid cancer; DFS: disease‐free survival; HGFM: high‐grade follicular cell‐derived malignancies.

In the present cohort, the 5‐year disease‐free survival was 59.4% and 44.7% in the HGFM and AG‐PTC groups, respectively (Figure 1). HR for univariate analysis was 0.53 (*p* = 0.073), whereas HR for multivariate analysis was 0.47 (*p* = 0.031), both of which were in favor of HGFM (Table 3). In other words, patients with HGFM were 53% less likely to have persistent or recurrent disease compared to those with AG‐PTC.

**TABLE 3:** Univariate and multivariate analyses of disease‐free survival.

	Univariate			Multivariate		
	HR	CI	*p*	HR	CI	*p*
HGFM	0.53	0.265–1.061	0.073	0.471	0.234–0.945	0.031
Age > =55	2.761	1.369–5.571	0.005	2.768	1.362–5.625	0.005
Male	2.213	1.125–4.355	0.021	2.236	1.128–4.432	0.021
Ki > = 10%	1.487	0.597–3.669	0.394	1.833	0.728–4.619	0.199
BRAF+	1.606	0.649–3.97	0.305	2.168	0.854–5.508	0.104
Percent > = 30%	1.4	0.668–2.931	0.372	1.641	0.757–3.56	0.21
VI	2.53	1.241–5.157	0.011	2.532	1.239–5.176	0.011
Partial biochemical	3.174	1.482–6.8	0.003	2.228	0.995–4.985	0.051
Partial structural	2.255	1.094–4.467	0.028	1.898	0.844–4.271	0.121
Tumor size > 3 cm	3.727	1.641–7.606	< 0.001	3.413	1.495–7.050	0.002
LVI	3.707	1.662–8.969	0.002	3.774	1.763–8.789	0.002
ETE	4.171	1.709–10.18	0.002	4.238	1.721–10.437	0.002
Solid	0.375	0.126–1.013	0.053	0.413	0.145–1.178	0.098
Tall cell	1.899	0.894–3.617	0.073	1.868	0.873–3.733	0.091

Abbreviations: AG‐PTC: aggressive‐papillary thyroid cancer; ETE: extrathyroidal extension; HGFM: high‐grade follicular cell‐derived malignancies; LVI: lymphovascular invasion; VI: vascular invasion.

In the multivariate cox regression analysis, the recurrence rate was 2‐fold higher in males than in females (HR: 2.236, confidence interval [CI] = 1.13–4.43, *p*‐value 0.021). The risk of recurrence was 2.8‐fold higher in patients aged ≥ 55 years than in younger patients. In addition, recurrence rates were significantly higher among patients with invasive histological features on surgical pathology, including vascular invasion (HR = 2.5, *p*‐value 0.011), tumor size > 3 cm (HR = 3.4, *p*‐value 0.002), lymphovascular invasion (HR = 3.8, *p*‐value 0.002), and extrathyroidal extension (HR = 4.2, *p*‐value 0.002) (Table 3).

## 4 Discussion

In this study, the prevalence of AG‐PTC and HGFM in the cohort was low at 2.2% and 1.9%, respectively. These are close to the rates reported in the literature for PDTC but lower than the rates reported for AG‐PTC [20, 21, 22]. In a recent multicenter study, the rates of AG‐PTC were around 13%, most of which were tall cell [23]. These are significantly higher than what we have reported and one of the possible reasons for this discrepancy is the fact that we included patients who were operated from 2010 unlike the referenced study here where they we included patients from 2020 onwards. It could be that the detection strategy for aggressive histology and recognition rate improved over the last 2 decades. After a mean follow‐up period of 4.52 +/− 5.04 and 4.89 +/− 4.89 years for AG‐PTC and HGFM, respectively, the rates of recurrent disease were found to be 29.9% (AG‐PTC) and 22.4% (HGFM), whereas the rates of persistent disease were 19.4% (AG‐PTC) and 15.5% (HGFM). These rates are significantly higher than those for well‐differentiated thyroid cancers. Ibrahimpasic et al. reported a recurrence rate of 18% in patients with PDTC, and 26% of patients had persistent disease [5]. The tall‐cell subtype in the present cohort was associated with a higher rate of recurrent/persistent disease; however, this association did not reach statistical significance. Coca‐Pelaz reported that the tall‐cell subtype is associated with more aggressive disease, and 20% of the tall cells are refractory to RAI [6]. Consistent with that in the present study, more aggressive tumors and worse prognoses have been associated with older age, larger tumors, and gross extrathyroidal extension [5, 6]. In the present study, certain features of invasiveness (T3/T4 stage, lymphovascular invasion, extrathyroidal extension and lymph node metastasis) were more common in the AG‐PTC group than in the HGFM group. These factors might account for the lower disease‐free survival rate among patients with AG‐PTC. Wu et al. examined a cohort of 365 individuals diagnosed with PTC with tall‐cell, discerning that positive surgical margins, nodal metastases, and a primary tumor dimension exceeding 3 cm serve as independent predictors for locoregional recurrence‐free survival [24]. The investigation by Kunte et al. into PDTC demonstrated that elevated tumor and nodal stages correlate with significantly worse outcomes, irrespective of the therapeutic methods applied [25].

Furthermore, Ki‐67 index is a prognostic marker in many types of tumors [14, 24, 25]. Volante et al. revealed that tumor necrosis and mitotic count in cases of solid/trabecular/insular carcinoma are the most relevant indicators for prognosis [26], which was consistent with the literature on the Ki‐67 index [16, 27, 28]. Tang reported a linear correlation between tumor size and Ki‐67 index with a cutoff value > 2.5% predicting more recurrence and mortality rates [17]. Additionally, the univariate analysis by Hescot et al. identified that possessing a mitotic count greater than 5 per 2 mm^2^ was a factor associated with an increased risk of disease recurrence in thyroid cancer patients [29]. In the present study, an increase in recurrence rates with a Ki‐67 index > 10% was observed; however, this did not reach statistical significance. This could be because of the small number of patients with documented Ki‐67 data or the low tested cutoff value. If a higher cutoff value had been used, positive results would probably have been achieved. Subgroup analysis revealed that patients with a Ki‐67 index ≥ 20% had a significantly higher rate of recurrent disease than those with a Ki‐67 index < 20%.

*BRAF* V600E mutation positivity was found in 16.7% of patients in the HGFM group, which is close to the rate reported earlier [30]. However, the positivity rate was much higher in the AG‐PTC cohort (84.6%) of the present study, as expected with the papillary nuclear atypia. Jin et al. identified that aggressive subtypes of Papillary Thyroid Carcinoma showed a higher incidence of BRAF mutations (89%) and a lower incidence of RAS mutations (3%) [31]. A previous study reported that *BRAF* V600E mutation is associated with aggressive disease and must be investigated in patients with small papillary thyroid carcinoma (< 1.5 cm) or papillary microcarcinoma (< 1 cm) to help identify high‐risk groups and initiate ideal treatment [32]. The present study did not show a relationship between the presence of *BRAF* mutation and the recurrence rate, possibly owing to the small number of patients with reported *BRAF* genetic testing because it was not routinely performed in all patients until recently. Despite the lack of statistical significance, there was a numerical increase in rates of persistent/recurrent disease among BRAF positive patients. This indicates that *BRAF* mutation is a promising prognostic marker for AG‐PTC. Furthermore, more genetic testing must be performed in a larger population of patients with advanced thyroid cancer to discover possible biomarkers associated with disease progression, evaluate prognosis accurately, and initiate better treatment plans.

Higher recurrence rates among patients with ≥ 30% tumor involvement were observed; however, this did not reach statistical significance, supporting recent studies reporting that even tumor involvement of > 10% can cause adverse outcomes. The mortality rate in the HGFM group of our cohort was relatively low at 1.8% compared with that in the large trial of patients with PDTC conducted by Ibrahimpasic et al., who reported a mortality rate of approximately 30% [5]. However, considering the relatively high rate of distant metastasis at presentation in the HGFM group, higher long‐term mortality rate is expected in this cohort of patients.

This study has some limitations. First, the retrospective nature of this study inevitably resulted in missing data. Furthermore, given the retrospective and longitudinal nature of this study, maintaining consistency in the diagnosis across pathology slides represents a significant challenge. To mitigate this, we strictly included cases with definitive diagnoses and excluded any with ambiguous pathology reports. In addition, BRAF and Ki‐67 data were documented in a relatively small number of patients because genetic testing was not routinely performed on all pathology slides at our center until recently. In addition, presence of BRAF mutation was tested using immunohistochemistry, which might not be as sensitive as direct sequencing. Evidence suggests that other late mutations, such as *TP53* [] and *TERT* mutations [30], in combination with those in *BRAF*, contribute more to an aggressive tumor type, and these mutations were minimally assessed in the present cohort. Further studies should investigate these mutations. Finally, the short follow‐up period might have underestimated the recurrence and mortality rates. Further studies with longer follow‐up periods should compare patients with consistent findings and results reported. Moreover, further studies with review of pathology slides could strengthen the results and ensure consistent diagnosis.

## 5 Conclusion

AG‐PTC and HGFM are aggressive thyroid tumors with high recurrence and persistence rates. Possible prognostic markers for predicting recurrent and persistent thyroid cancers and guiding therapy include tumor size > 3 cm, lymphovascular invasion, vascular invasion, extrathyroidal extension, response to primary therapy, and the proliferative index Ki‐67. AG‐PTC and HGFM should be recognized and followed up more closely with intense treatment strategies based on prognostic markers.

## Author Contributions

S.R. wrote the proposal, collected the data, and wrote the manuscript, M.T. originated the idea, supervised the process, and revised the manuscript, M.A. wrote and reviewed the manuscript, S.B. and M.F. performed the analysis, R.P. originated the idea and supervised the process.

## Ethics Statement

This study was approved by the Medical‐Bioethics Research Ethics Committee (REC) of the Integrated Health and Social Services Network for West‐Central Montreal Research Ethics Board (REB), project number 2020‐1902. Informed consent from participants was not required and deemed unnecessary according to the national regulations as this was a retrospective study.

## Consent

The authors have nothing to report.

## Conflicts of Interest

The authors declare no conflicts of interest.

## Data Availability

All data generated or analyzed during the study are included in the article. The datasets are available from the corresponding author on reasonable request.
